# Risk of cardiometabolic outcomes among women with a history of pelvic inflammatory disease: a retrospective matched cohort study from the UK

**DOI:** 10.1186/s12905-023-02214-5

**Published:** 2023-02-23

**Authors:** Kelvin Okoth, G. Neil Thomas, Krishnarajah Nirantharakumar, Nicola J. Adderley

**Affiliations:** 1 Institute of Applied Health Research, University of Birmingham, Birmingham, UK; 2 Institute of Metabolism and Systems Research, University of Birmingham, Birmingham, UK; 3 Centre for Endocrinology, Diabetes and Metabolism, Birmingham Health Partners, Birmingham, UK

**Keywords:** Pelvic inflammatory disease, Cardiovascular disease, Hypertension, Type 2 diabetes mellitus

## Abstract

**Introduction:**

To describe the incidence and prevalence of pelvic inflammatory disease (PID) and to estimate the risk of cardiometabolic outcomes among women with PID compared to women without PID.

**Methods:**

A UK retrospective matched cohort study using data from The Health Improvement Network. To assess cardiometabolic risk, women (aged ≥ 16 years) with PID were compared to matched controls without PID. Annual prevalence and incidence of PID (1998–2017) were estimated among women aged 16–50 years using annual cross-sectional and cohort analyses, respectively. Adjusted hazard ratios (aHR) and 95% CI for cardiometabolic outcomes were estimated using Cox proportional hazards models. The primary outcome was composite cardiovascular disease (CVD) and its subtypes, including ischaemic heart disease (IHD), heart failure (HF) and cerebrovascular disease. Secondary outcomes were hypertension, and type 2 diabetes mellitus (T2DM).

**Results:**

Among the 715 recorded composite CVD events, the crude incidence rate per 1000 person-years was 1.5 among women with history of PID compared to 1.3 in matched controls. Compared to women without PID (N = 73,769), the aHRs for cardiometabolic outcomes among women with PID (N = 19,804) were: composite CVD 1.10 (95% CI 0.93–1.30); IHD 1.19 (95% CI 0.93–1.53); cerebrovascular disease 1.13 (95% CI 0.90–1.43); HF 0.92 (95% CI 0.62–1.35) hypertension 1.10 (95% CI 1.01–1.20); and T2DM 1.25 (95% CI 1.09–1.43). The prevalence (per 10,000 population) of PID was 396.5 in 1998 and 237 in 2017. The incidence (per 10,000 person-years) of PID was 32.4 in 1998 and 7.9 in 2017.

**Conclusion:**

There was no excess risk of composite CVD or its subtypes among women with history of PID compared to matched controls. Findings from our study suggest that history of PID was associated with an increased risk of hypertension and type 2 diabetes mellitus, two major risk factors for CVD. Additional studies are required to support these findings.

**Supplementary Information:**

The online version contains supplementary material available at 10.1186/s12905-023-02214-5.

## Background

Cardiovascular disease (CVD) is a major public health burden with the most recent estimates from Europe revealing that it accounted for a third of all premature deaths among women [1]. Despite a notable reduction in CVD burden in recent decades, there is potential for a resurgence. Analysis of sex-aggregated data unmasks differential trends in CVD mortality, with rates in older age groups (> 65 years) showing a steady decline, while rates in younger adults (< 55 years) show stagnation [2]. With up to 20% of CVD unexplained by traditional risk factors, non-traditional risk factors for CVD are gaining prominence [3]. Female reproductive complications are associated with cardiovascular outcomes in later life [4]. However, evidence on the association between infections of the female reproductive tract and CVD is limited. Findings from nascent literature suggest an increased risk of cardiovascular outcomes among women with a history of pelvic inflammatory disease [5, 6]. However, these studies had several limitations, including a misleading case definition, a short duration of follow-up, and lack of adjustment for key confounders.

PID refers to infection of the female upper genital tract. The causative microorganisms are polymicrobial with *Chlamydia trachomatis* and *Neisseria gonorrhoea* accounting for 35% of the cases in the UK [7]. Infectious agents may aid in the development of cardiometabolic outcomes through the direct assault of the vasculature or indirect systemic effects of response to infection [8]. The present study estimated the burden of PID among UK women and explored the association between history of PID and risk of cardiometabolic outcomes.

## Methods

### Study design

Serial cross-sectional studies were carried out on 1st January each year from 1998 to 2017 to estimate the prevalence of PID. To estimate the annual incidence rate, a series of cohort studies were conducted over the same period. A population-based retrospective cohort study (1995–2018) was conducted to assess risk of long-term cardiometabolic outcomes.

### Data source

This work used de-identified data provided by patients as a part of their routine primary care. Study data was extracted from IQVIA Medical Research Data (IMRD-UK), which incorporates data from The Health Improvement Network (THIN). The Health Improvement Network is one of the largest UK primary care databases containing electronic health records from England, Wales, Scotland, and Northern Ireland. By 2019, more than 800 practices spread throughout the UK had contributed data to THIN [9]. Validation studies have demonstrated that THIN is representative of the UK with regard to demographics, mortality (adjusted for demographics and deprivation) and prevalence of major conditions [10–12]. Participating practices record patient data using Vision software, an electronic health records system. Practices were eligible for inclusion from the later of one year after the date on which the practice met acceptable mortality reporting (a quality assurance standard and one year after the practice began to use the Vision system, to maximise data quality and recording).

### Study population

For incidence and prevalence, female patients aged 16–50 years and registered with an eligible practice for ≥ 1 year before study entry (to ensure documentation of all-important baseline covariates) were eligible for inclusion [13]. For the cardiometabolic outcomes study, women aged ≥ 16 years at baseline were included in the study. The study period was 1st January 1995–31st December 2018. Participants were eligible to enter the study at the latest of their 16th birthday, study start date and one year after joining the practice [13].

### Exposure

Women with PID (exposed group) were matched with up to four randomly selected women without PID (unexposed group). The exposed and unexposed groups were matched by age (± one year), general practice, and body mass index, BMI (± 2 kg/m^2^). Diagnoses of PID were identified using UK primary care diagnostic codes (Read codes). The present study adopted the version of the PID Read code list developed by French and colleagues who grouped diagnostic codes for PID in UK primary care into three separate categories, defined as “definite”, “probable” and “possible” [14]. The present study combined “definite” and “probable” into a single category; only participants who fulfilled this definition of PID were included in the study. A search of the Read code dictionary did not reveal any additional codes beyond the list developed by French and colleagues. Patients with a record of “possible” PID were excluded from the study.

### Follow-up period

The date of diagnosis of PID served as the index date for exposed patients. Unexposed patients were assigned the same index date as their corresponding exposed patient in order to mitigate immortal time bias [15]. Each exposed participant and matched controls were followed up from the index to the exit date. The exit date was the earliest of (1) the outcome, (2) death, (3) study end date, (4) date of leaving the general practice or when the general practice stopped contributing to the database [13].

### Outcomes

The primary outcomes were an incident diagnosis of composite CVD and the following individual cardiovascular outcomes: heart failure, cerebrovascular accident (stroke and transient ischaemic attack (TIA)), and IHD. Secondary outcomes were an incident diagnosis of type 2 diabetes mellitus and hypertension. Patients with a record of any CVD at baseline were excluded from the analysis for primary outcomes; patients with a record of type 2 diabetes or hypertension at baseline were excluded from the respective analyses. For example, patients with a record for type 2 diabetes mellitus at baselines were excluded from the analyses examining the association between PID and risk of type 2 diabetes mellitus. The Read codes used in the presented study were selected through a meticulous process similar to the methodology proposed by Davé and Peterson and Watson et al. [16, 17]. First, a list of relevant medical terms relating to the outcomes were developed. Second, the description and alpha-numeric fields (columns) of the Read code dictionary were searched for relevant codes using the medical terms identified in the first step. Third we compared the codes identified in the previous step to codes published in online Read code repositories (caliberresearch.org, clinicalcodes.org, Cambridge code lists index) [18–20], and codes published in supplemental material of existing literature [21, 22]. Finally, we consulted UK clinicians to develop the final set of codes to be used in the study. All the outcomes in the present study are part of the UK quality and outcomes framework (QOF), a pay for performance scheme. The QOF was created to improve chronic disease management by financially rewarding primary care practices for providing interventions linked to improved health outcomes [23]. Chronic conditions that fall under the QOF domains are well-recorded in UK general practices. Validation studies have demonstrated that the prevalence of chronic conditions in THIN database is comparable to national estimates [10–12].

### Potential confounders

The study included the following potential confounders: age, Townsend deprivation quintile, BMI (categorised as < 18.5 kg/m^2^ (underweight, 18.5–25 kg/m^2^ (normal weight), 25–30 kg/m^2^ (overweight), > 30 kg/m^2^ (obese) and missing), smoking status (categorised as non-smokers, smokers, ex-smokers and missing), lipid medication (current users, with a record of a prescription within 60 days prior to index date), diabetes mellitus, hypertension, contraceptive use (current users, defined as those prescribed combined oral contraceptive pills within the last 365 days prior to cohort entry), and reproductive conditions (gestational diabetes mellitus, pre-term delivery, miscarriage, pre-eclampsia, stillbirths, polycystic ovary syndrome, endometriosis). For each of the covariates, the most recently recorded measurement before study entry was used [13, 24].

### Analysis

#### Prevalence and incidence

For annual point prevalence, the proportion of eligible females with any record ever of PID was calculated on 1st January each year from 1998 to 2017. Crude incidence rates every year from 1998 to 2017 were calculated by dividing the number of newly diagnosed females (numerator) by the total number of person-years at risk (denominator) for the given year. Incidence rates by Townsend quintiles of deprivation and age-specific categories were also calculated using data for the whole period (1998–2017) [13, 24].

#### Cardiometabolic outcomes

Participant characteristics at baseline were reported using mean (SD) or median (IQR) for continuous variables and numbers (%) for categorical data. Both unadjusted and adjusted Cox proportional hazards models were used to estimate the hazard ratios (HR) and 95% CI of incident cardiometabolic conditions among women exposed to PID compared to those unexposed to PID. In the multivariable models, adjustments were made for age, smoking status, hormonal contraceptive use, lipid profile, BMI, pre-eclampsia, gestational diabetes, pregnancy loss, and endometriosis [13, 24]. For each model, the proportional hazards assumption was checked using the Schoenfeld residuals test followed by a graphical confirmation using the log–log survival curves. Statistical significance was set at 0.05. All analyses were conducted in Stata SE version 17.0.

#### Sensitivity analysis

We hypothesized that women diagnosed with PID may have frequent primary care contacts after the index date therefore, some degree of surveillance bias cannot be ruled out. Surveillance bias is a type of bias in which the exposure group has a higher or lower chance of being screened for the outcome than the comparator group or the general population [25]. In a sensitivity analysis, a two-year lag period was introduced after the index date to investigate the possibility of surveillance bias.

## Results

### Prevalence and incidence

**Fig. 1:**
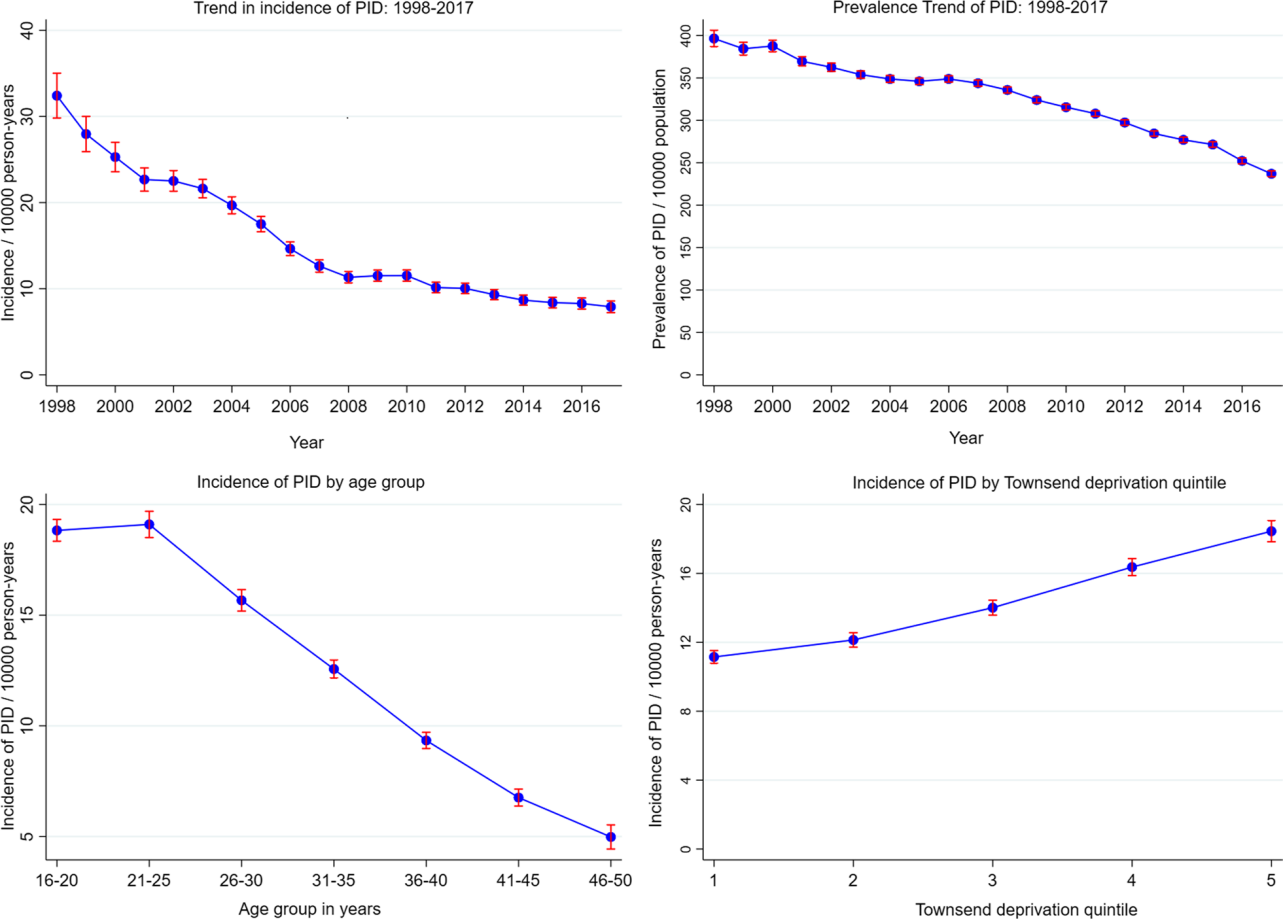
The incidence and prevalence of pelvic inflammatory disease among UK women: 1998–2017. Townsend quintiles: 1 – least deprived, 5 – most deprived

Overall, both the annual prevalence and incidence of PID among women in the UK decreased throughout the twenty-year period of study (Fig. 1, Additional file 1: Tables S1 and S2). The prevalence (per 10,000 population) of PID decreased from 396.5 in 1998 to 237.0 in 2017. The incidence (per 10,000 person-years) of PID decreased from 32.4 per in 1998 to 7.9 in 2017.

The incidence of PID decreased with increasing age. The incidence of PID was highest among women aged 20–24 years (19.1 per 10,000 person-years at risk) and lowest among women aged 45–50 years (5.0 per 10,000 person-years at risk) (Fig. 1, Additional file 1: Table S3). The incidence of PID was highest among women from the most deprived population (18.5 per 10,000) and lowest among the least deprived population (11.2 per 10,000 person-years at risk) (Fig. 1 and Additional file 1: Table S4).

### Cardiometabolic outcomes

Additional file 1: Figure S1 presents the study participants flow chart. After application of inclusion and exclusion criteria, 19,804 (21%) and 73,769 (79%) women were included in the exposed and comparator groups, respectively. The baseline characteristics of the study participants are summarised in Table 1. Women with a history of PID compared to those without a history of PID were more likely to: be from the most deprived quintile (16.1% vs. 13.3%), be smokers (33.3% vs. 22.3%), have a history of miscarriage (12.5% vs. 6.4%), have a diagnosis of polycystic ovary syndrome (3.3% vs. 1.9%), be diagnosed with endometriosis (3.2% vs. 0.9%), and have a current prescription for combined oral contraceptive pills (10.3% vs. 7.9%).

**Table 1:** Baseline demographic, lifestyle, reproductive and medical characteristics among women with pelvic inflammatory disease (PID) and those without PID

Characteristic	Pelvic inflammatory disease (n = 19,804)	Without pelvic inflammatory disease (n = 73,769)
	n (%)	n (%)
*Age (years)*		
< 20	3319 (16.8)	14,315 (19.4)
21–30	6865(34.7)	24,942 (33.8)
31–40	5786 (29.2)	21,027 (28.5)
41–50	2368 (12.0)	8325 (11.3)
> 50	1466 (7.4)	5160 (7.0)
*BMI categories (kg/m*^*2*^*)*		
< 18.5	779 (3.9)	2569 (3.5)
18.5–25	8374 (42.3)	29,109 (39.5)
25–30	3880 (19.6)	12,808 (17.4)
> 30	2858 (14.4)	9771 (13.3)
Missing or implausible	3913 (19.8)	19,512 (26.5)
*Townsend deprivation quintile*		
1 (least deprived)	3361 (17.0)	14,744 (20.0)
2	3179 (16.1)	12,815 (17.4)
3	3765 (19.0)	13,807 (18.7)
4	3862 (19.5)	13,380 (18.1)
5 (most deprived)	3178 (16.1)	9843 (13.3)
Missing	2459 (12.4)	9180 (12.4)
*Smoking status*		
Non-smokers	9212 (46.5)	39,957 (54.2)
Ex-smokers	2826 (14.3)	8368 (11.3)
Smokers	6599 (33.3)	16,462 (22.3)
Missing	1167 (5.9)	8982 (12.2)
Current lipid	318 (1.6)	1021 (1.4)
Hypertension	803 (4.1)	2522 (3.4)
Diabetes	290 (1.5)	861 (1.2)
*Reproductive history*		
Gestational diabetes	126 (0.6)	273 (0.4)
Pre-term delivery	163 (0.8)	458 (0.6)
Miscarriage	2471 (12.5)	4738 (6.4)
Stillbirth	80 (0.4)	195 (0.3)
Pre-eclampsia	92 (0.5)	281 (0.4)
Polycystic ovary syndrome	643 (3.3)	1405 (1.9)
Endometriosis	638 (3.2)	672 (0.9)
Current combined oral contraceptive pills	2043 (10.3)	5848 (7.9)

*BMI*, Body mass index, *kg*, kilograms, *m*, metres

**Fig. 2:**
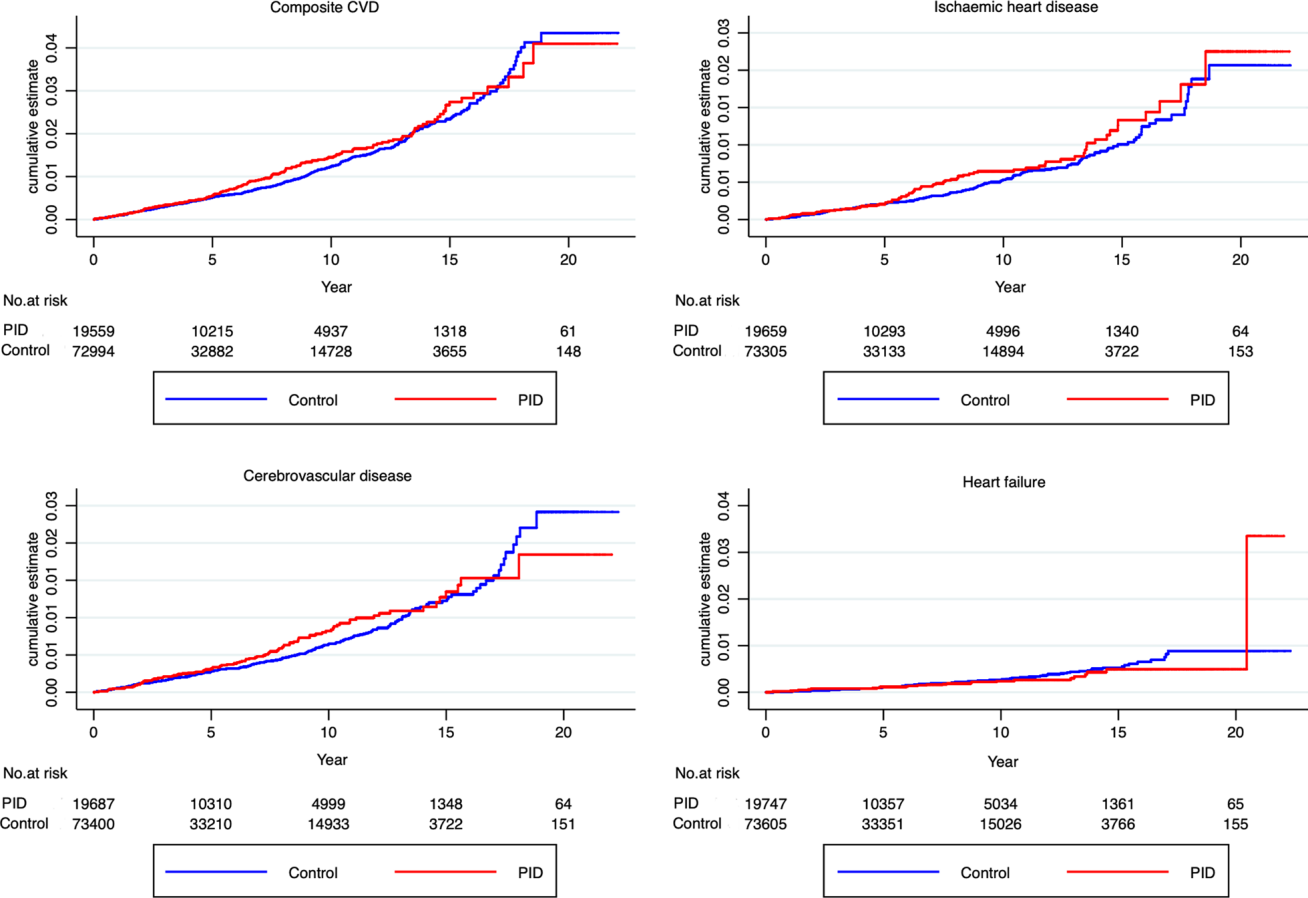
Cumulative hazard of primary cardiometabolic outcomes among women with a history of PID (exposed) and those without a history of PID (unexposed)

The incidence rates (per 1000 person-years) of composite CVD among women with a history of PID compared to those without a history of PID were 1.5 and 1.3, respectively (Table 2). The median (inter-quartile range) follow-up time was 4.5 (1.7–9.0) years. In the unadjusted model, the aHR for composite CVD was 1.11 (95% CI, 0.94–1.31). The multivariable model included demographic, lifestyle, medical (hypertension, diabetes mellitus) and reproductive characteristics. Adjustment did not impact the hazard ratio of composite CVD (aHR 1.10; 95% CI 0.93–1.30) (Table 2, Fig. 2).

**Table 2:** Incidence rates and hazard ratios for composite cardiovascular disease (CVD) and CVD subtypes for women with a history of PID compared to those without a history of PID

	Composite CVD		Ischaemic heart disease		Cerebrovascular disease		Heart failure	
	Exposed	Unexposed	Exposed	Unexposed	Exposed	Unexposed	Exposed	Unexposed
Population	19,559	72,994	19,659	73,305	19,687	73,400	19,747	73,605
Events, n (%)	184	531	86	228	100	283	33	120
Person-years	126,220.7	413,438.4	127,279.9	416,644.2	127,453.4	417,432.6	128,107.8	419,306.3
Crude incidence rate/1000 person years	1.5	1.3	0.7	0.5	0.8	0.7	0.3	0.3
Crude HR (95% CI)	1.11 (0.94–1.31)		1.20 (0.93–1.54)		1.13 (0.90–1.42)		0.87 (0.59–1.28)	
*P*-value	0.238		0.154		0.289		0.486	
Adjusted HR (95% CI)	1.10 (0.93–1.30)		1.19 (0.93–1.53)		1.13 (0.90–1.43)		0.92 (0.62–1.35)	
*P*-value	0.275		0.176		0.284		0.671	

Model adjusted for age, Townsend deprivation quintile, BMI, smoking status, lipid-lowering medication (current users, with a record of a prescription within 60 days prior to index date), diabetes mellitus hypertension contraceptive use (current users, defined as those prescribed combined oral contraceptive pills within the last 365 days prior to cohort entry) and reproductive conditions (premature delivery, miscarriage, stillbirths, gestational diabetes mellitus, polycystic ovary syndrome, pre-eclampsia, endometriosis

*CVD*, composite CVD, *HR*, Hazard ratio,

On examination of CVD subtypes, compared to women without history of PID, the aHR for CVD subtypes in women with history of PID were: 1.19 (95% CI 0.93–1.53) for IHD; 1.13 (95% CI 0.90–1.43) for cerebrovascular disease; 0.92 (95% CI 0.62–1.35) for heart failure (Table 2, Fig. 2).

**Fig. 3:**
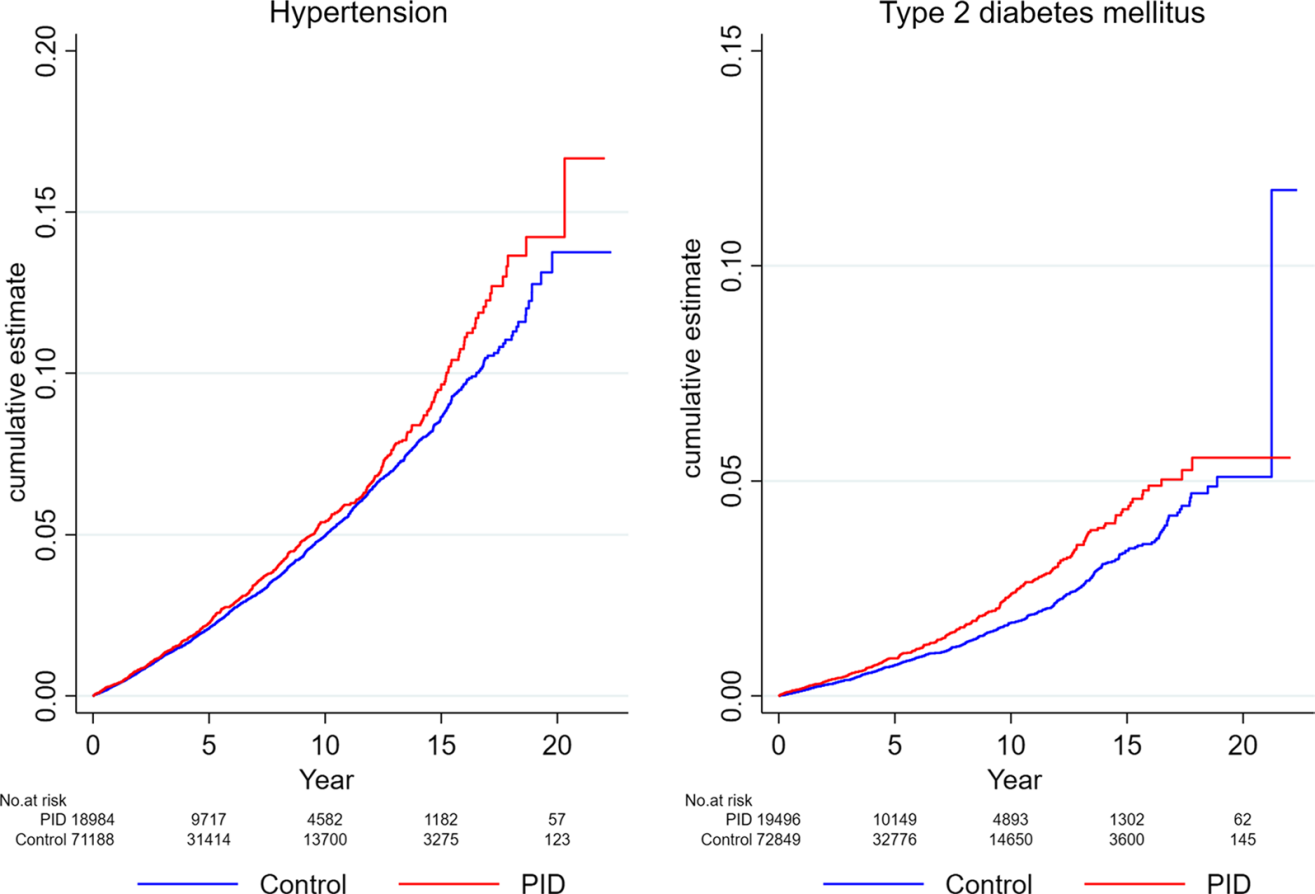
Cumulative hazard of secondary cardiometabolic outcomes among women with a history of PID (exposed) and those without a history of PID (unexposed)

In the crude model, the HR for hypertension was marginally increased (HR 1.09; 95% CI 1.00–1.19) among women with history of PID compared to controls without history of PID. In the adjusted model, the aHR for hypertension was 1.11 (95% CI, 1.01–1.21; *P* = 0.023) among women with history of PID compared to those without history of PID (Table 3 and Fig. 3). The crude HR for type 2 diabetes among women with history of PID compared controls without history of PID was 1.31 (95% CI, 1.14–1.50). In the adjusted model the HR was slightly attenuated but remained significant (aHR 1.25; 95% CI 1.09–1.43) (Table 3 and Fig. 3).

**Table 3:** Incidence rates and hazard ratios for hypertension and type 2 diabetes mellitus for women with a history of pelvic inflammatory disease (PID) compared to those without a history of PID

	Hypertension		Type 2 diabetes mellitus	
	Exposed	Unexposed	Exposed	Unexposed
Population	18,984	71,188	19,496	72,849
Events, n (%)	658	1942	294	720
Person-years	119,971.3	394,438.7	125,431.3	411,909.9
Crude incidence rate/1000 person years	5.5	4.9	2.3	1.7
Crude HR (95% CI)	1.09 (1.00–1.19)		1.31 (1.14–1.50)	
*P*-value	0.051		< 0.001	
Adjusted HR (95% CI)	1.10 (1.01–1.20)*		1.25 (1.09–1.43)^#^	
*P*-value	0.038		< 0.002	

Model adjusted for age, Townsend deprivation quintiles, BMI, smoking status, lipid-lowering medication (current users, with a record of a prescription within 60 days prior to index date) contraceptive use (current users, defined as those prescribed combined oral contraceptive pills within the last 365 days prior to cohort entry) and reproductive conditions (premature delivery, miscarriage, stillbirths, gestational diabetes mellitus, polycystic ovary syndrome, pre-eclampsia, endometriosis)

*CVD*, composite CVD, *HR*, hazard ratio

*Model adjusted for diabetes mellitus, ^#^Model adjusted for hypertension

In a sensitivity analysis, the introduction of a two-year lag period to investigate the possibility of surveillance bias had minimal impact on the results. The adjusted HRs for cardiometabolic outcomes in women with history of PID compared to matched controls were: 1.13 (95% CI 0.93–1.36) for composite CVD; 1.20 (95% CI 0.91–1.58) for IHD; 1.18 (95% CI 0.91–1.53) for cerebrovascular disease; 0.64 (95% CI 0.40–1.04) for heart failure 1.11 (95% CI 1.00–1.23) for hypertension; and 1.26 (95% CI 1.08–1.47) for type 2 diabetes mellitus (Additional file 1: Tables S5 and S6).

## Discussion

This study explored risk of cardiometabolic outcomes among women with a history of PID compared to a matched comparator group of women without a history of PID. We found no evidence of association between a history of PID and risk of composite CVD or subtypes of CVD. The evidence from our study suggests that women with a history of PID were at a significantly higher risk of hypertension and T2DM.

The main strengths of our study include a large sample size from a population representative of the general UK population, a long duration of follow-up and the availability of information on known and potential confounders.

Our study has several limitations. We lacked information on the mode of diagnosis; therefore, we could not distinguish cases diagnosed through laparoscopy or endometrial biopsy from those diagnosed by other means. Diagnoses based on clinical findings are practicable, cost-effective and capture a vast majority of PID cases as demonstrated by several studies [26–28]. Although diagnoses based on clinical findings captures the vast majority of cases, there are no studies that have examined the validity of PID diagnoses in UK primary care databases. Since CVD is part of the Quality and Outcomes Framework, this is well recorded in UK primary care. Second, the case definition used in our cohort study relied on physician assigned codes for PID. We used a restricted definition for PID in our analyses to minimise the use of non-specific symptom codes to diagnose PID. Consequently, the true incidence and prevalence estimates of PID may be underestimated. Third, the exposed cohort was composed of only clinically diagnosed PID cases; there is therefore a likelihood for the inclusion of subclinical PID cases in the unexposed group. However, any observed associations are likely to be biased towards the null. Fourth, ethnicity data is not well-recorded in the THIN database, therefore, it was not included in the adjusted analyses. Fifth, because we made no adjustments for multiple comparisons, it is possible that the significant results observed in the study may be affected by type 1 error. Sixth, eligible patients (exposed and corresponding matched controls) were identified using the Data Extraction for Epidemiological Research (DExtER) tool [29]. Controls were defined as patients who did not have a record of PID at the index date or at any time-point afterward until the end of study. The use of future exposure information to define cohort membership at baseline may lead to bias [30]. However, since the incidence of PID is low, the effect estimates are unlikely to be affected.

Three studies examined the incidence rate and demographic characteristics of PID in the UK primary care setting [14, 31, 32]. French and colleagues reported a decline in the incidence rate of PID between 2000 and 2008 among women aged 15–44 years [14]. Likewise, reports from Public Health England reported a decline in rates between 2000 and 2011 [32]. The drivers behind the decline in rates of PID in the UK are not entirely clear. Improved testing technology and increased screening rates (national chlamydia screening programme in 2002) for chlamydia infection may have contributed to early treatment before the onset of PID [33]. In addition, a negative test on chlamydia screening may have contributed to a reduction in the coding for PID cases [33]. Our findings are consistent with previous UK studies which reported higher rates of PID among young persons aged 16–24 and those from lower socioeconomic groups. A major risk factor for PID infection is sexual behaviour. National UK surveys on sexual attitudes and lifestyles have consistently shown that younger individuals are more likely to have multiple partners and engage in risky sexual behaviour [34]. A lower index of deprivation may be a surrogate marker for sexual behaviour [35].

A search of the literature suggests that all the previously published studies evaluating the risk of cardiovascular outcomes among women with a history of PID were from Taiwan [5, 6, 36]. The Taiwanese studies noted that compared to women without history of PID, women with history of PID were at a significantly higher risk of myocardial infarction and ischaemic stroke but at a significantly lower risk of intracerebral haemorrhage. Methodological differences may explain the discordant findings between the present study and the Taiwanese studies. Foremost, the Taiwanese studies relied on data from administrative health databases which are typically designed for financial reimbursements and lacked additional health data on potential confounders. The present study adjusted for these missing variables. Also, in the Taiwanese studies, the lax case definition for PID that included ICD-9 codes for infections of the lower genital tract may have been misleading [37]. In addition, the because the study population is young a lower number of CVD events were recorded.

To the best of our knowledge, this is the first study to show a temporal association between an increased risk of hypertension and diabetes mellitus among women with a history of PID compared to women without PID. The exact mechanisms explaining the association between infections and elevated cardiometabolic risk are uncertain. One potential mechanism includes direct invasion of arterial vasculature [8]. Several studies have identified Chlamydia trachomatis in cardiovascular tissue suggesting that it may have a role cardiovascular disease development through local effects [38–40]. A second potential mechanism involves systemic responses to infection (indirect) effects. The molecular components of pathogens may be structurally similar (molecular mimicry) to host proteins resulting in a cross-reaction as the immune system recognises host proteins as foreign [41]. Systemic response to infections involves the release of cytokines and other acute phase reactant proteins (CRP). An increase in pro-inflammatory cytokines and acute phase proteins promotes cardiovascular disease via increased oxidative stress, impairment of endothelial nitric oxidase synthase, insulin resistance induction of endothelial cell apoptosis, increased uptake of low-density lipoproteins by macrophages, and adherence of monocytes into the arterial wall [42–44].

History of PID may serve as a marker for the future development of hypertension and diabetes mellitus (type 2), two prominent risk factors for CVD. The integration of the female reproductive history into routine primary care consultations may impact CVD risk factor screening [45]. Areas that warrant further research include: the variability in cardiometabolic risk based on the type of microbe causing PID or severity of PID.

## Conclusion

This study found no evidence of excess risk of composite CVD or its subtypes among women with history of PID compared to matched controls. Findings from our study suggests that history of PID was associated with an increased risk of hypertension and type 2 diabetes mellitus, two major risk factors for CVD. Additional studies are required to support these findings.

## Supplementary Information

**Additional file 1**: **Table S1**. Incidence rate of pelvic inflammatory disease among women aged 16–50 years: 1998–2017. **Table S2**. Prevalence of pelvic inflammatory disease among women aged 16–50 years: 1998–2017. **Table S3**. Incidence of pelvic inflammatory disease by age categories. **Table S4**. Incidence of PID among women aged 16–50 years by Townsend deprivation quintiles. **Fig. S1**. Study participant flow chart. **Table S5**. (Sensitivity analyses): Incidence rates and hazard ratios for composite cardiovascular disease (CVD) and CVD subtypes for women with a history of PID compared to controls. **Table S6**. (Sensitivity analyses): Incidence rates and hazard ratios for hypertension and type 2 diabetes mellitus for women with a history of pelvic inflammatory disease (PID) compared to controls.

## Data Availability

All data relevant to the study are included in the article or uploaded as supplementary information. All relevant data are within the paper and its supporting information files.
