# Supplementary material for: Risk of cardiometabolic outcomes among women with a history of pelvic inflammatory disease: a retrospective matched cohort study from the UK
Source: BMC Womens Health. 2023 Feb 23;23:80. doi: 10.1186/s12905-023-02214-5 (PMC9948336; doi:10.1186/s12905-023-02214-5)
Supplement: Supplementary file 1 — Additional file 1: Table S1. Incidence rate of pelvic inflammatory disease among women aged 16–50 years: 1998–2017. Table S2. Prevalence of pelvic inflammatory disease among women aged 16–50 years: 1998–2017. Table S3. Incidence of pelvic inflammatory disease by age categories. Table S4. Incidence of PID among women aged 16–50 years by Townsend deprivation quintiles. Fig. S1. Study participant flow chart. Table S5. (Sensitivity analyses): Incidence rates and hazard ratios for composite cardiovascular disease (CVD) and CVD subtypes for women with a history of PID compared to controls. Table S6. (Sensitivity analyses): Incidence rates and hazard ratios for hypertension and type 2 diabetes mellitus for women with a history of pelvic inflammatory disease (PID) compared to controls. [file 12905_2023_2214_MOESM1_ESM.docx]

**Additional file:**

Table S1: Incidence rate of pelvic inflammatory disease among women aged 16-50 years: 1998 -2017

| Year | No. of incident cases | Person-years | Incidence rate/10,000 person-years |
| --- | --- | --- | --- |
| 1998 | 596 | 183887.5 | 32.4 |
| 1999 | 720 | 257534.3 | 28.0 |
| 2000 | 839 | 331807.8 | 25.3 |
| 2001 | 1089 | 480284.6 | 22.7 |
| 2002 | 1356 | 602413.4 | 22.5 |
| 2003 | 1564 | 723199.5 | 21.6 |
| 2004 | 1531 | 777968.6 | 19.7 |
| 2005 | 1489 | 850801.9 | 17.5 |
| 2006 | 1318 | 900047.6 | 14.6 |
| 2007 | 1179 | 933595 | 12.6 |
| 2008 | 1119 | 988257.1 | 11.3 |
| 2009 | 1193 | 1035927.7 | 11.5 |
| 2010 | 1187 | 1030002.1 | 11.5 |
| 2011 | 1049 | 1034079.8 | 10.1 |
| 2012 | 1070 | 1065746 | 10.0 |
| 2013 | 970 | 1041861.9 | 9.3 |
| 2014 | 842 | 970712.1 | 8.7 |
| 2015 | 721 | 859902.1 | 8.4 |
| 2016 | 614 | 741442.6 | 8.3 |
| 2017 | 526 | 665698.1 | 7.9 |

Table S2: Prevalence of pelvic inflammatory disease among women aged 16-50 years: 1998-2017

| Year | No. of patients with PID | Population | Prevalence/10,000 population |
| --- | --- | --- | --- |
| 1998 | 6210 | 156630 | 396.5 |
| 1999 | 9480 | 246598 | 384.4 |
| 2000 | 11889 | 306733 | 387.6 |
| 2001 | 17287 | 467696 | 369.6 |
| 2002 | 20442 | 563755 | 362.6 |
| 2003 | 24931 | 704436 | 353.9 |
| 2004 | 27388 | 785251 | 348.8 |
| 2005 | 29077 | 840414 | 346.0 |
| 2006 | 32189 | 922729 | 348.9 |
| 2007 | 32967 | 958980 | 343.8 |
| 2008 | 33399 | 994539 | 335.8 |
| 2009 | 34462 | 1063695 | 324.0 |
| 2010 | 33973 | 1076777 | 315.5 |
| 2011 | 32851 | 1066644 | 308.0 |
| 2012 | 32185 | 1082059 | 297.4 |
| 2013 | 31413 | 1104362 | 284.4 |
| 2014 | 28576 | 1031610 | 277.0 |
| 2015 | 25977 | 956722 | 271.5 |
| 2016 | 20220 | 801646 | 252.2 |
| 2017 | 17216 | 726315 | 237.0 |

Table S3: Incidence of pelvic inflammatory disease by age categories

| Age categories | No. of incident cases | Person-years | Incidence rate/10,000 person-years |
| --- | --- | --- | --- |
| 16-19 years | 5664 | 3007871.8 | 18.8 |
| 20-24 years | 3976 | 2081580.1 | 19.1 |
| 25-29 years | 4041 | 2579121.8 | 15.7 |
| 30-34 years | 3684 | 2932162.5 | 12.6 |
| 35-39 years | 2475 | 2649987 | 9.3 |
| 40-44 years | 1196 | 1769741.3 | 6.8 |
| 45-50 years | 321 | 644913.5 | 5.0 |

Table S4: Incidence of PID among women aged 16-50 years by Townsend deprivation quintiles

| Townsend quintile of deprivation | No. of incident cases | Person-years | Incidence rate/10,000 person-years |
| --- | --- | --- | --- |
| 1 (least deprived) | 3465 | 3107992.5 | 11.2 |
| 2 | 3258 | 2684137 | 12.1 |
| 3 | 3980 | 2840628 | 14.0 |
| 4 | 4217 | 2576176.3 | 16.4 |
| 5 (most deprived) | 3489 | 1891168.3 | 18.5 |

Table S5 (Sensitivity analyses): Incidence rates and hazard ratios for composite cardiovascular disease (CVD) and CVD subtypes for women with a history of PID compared to controls

|  | Composite CVD | | Ischaemic Heart Disease | | Cerebrovascular disease | | Heart failure | |
| --- | --- | --- | --- | --- | --- | --- | --- | --- |
|  | Exposed | Unexposed | Exposed | Unexposed | Exposed | Unexposed | Exposed | Unexposed |
| Population | 14966 | 51933 | 15062 | 52235 | 15074 | 52319 | 15130 | 52498 |
| Events, n (%) | 150 | 409 | 72 | 187 | 80 | 210 | 20 | 100 |
| Person-years | 91,859.8 | 290,272.0 | 92,729.8 | 292,862.3 | 92,859.4 | 293,478.5 | 93,402.0 | 294,966.5 |
| Crude incidence rate/1000 person years | 1.6 | 1.4 | 0.8 | 0.6 | 0.9 | 0.7 | 0.2 | 0.3 |
| Crude HR (95% CI) | 1.14 (0.94-1.37) | | 1.19 (0.91-1.57) | | 1.18 (0.91-1.53) | | 0.61 (0.38- 0.99) | |
| P-value | 0.183 | | 0.204 | | 0.210 | | 0.047 | |
| Adjusted HR (95% CI) | 1.13 (0.93-1.36) | | 1.20 (0.91-1.58) | | 1.18 (0.91-1.53) | | 0.64 (0.40-1.04) | |
| P-value | 0.213 | | 0.197 | | 0.220 | | 0.072 | |

CVD = composite CVD, HR= Hazard ratio.

Model adjusted for age, Townsend deprivation quintiles, BMI, smoking status, lipid-lowering medication (current users, with a record of a prescription within 60 days prior to index date), diabetes mellitus hypertension contraceptive use (current users, defined as those prescribed combined oral contraceptive pills within the last 365 days prior to cohort entry) and reproductive conditions (premature delivery, miscarriage, stillbirths, gestational diabetes mellitus, polycystic ovary syndrome, pre-eclampsia, endometriosis).

Table S6 (Sensitivity analyses): Incidence rates and hazard ratios for hypertension and type 2 diabetes mellitus for women with a history of pelvic inflammatory disease (PID) compared to controls

|  | Hypertension | | Type 2 diabetes mellitus | |
| --- | --- | --- | --- | --- |
|  | Exposed | Unexposed | Exposed | Unexposed |
| Population | 14,438 | 50,292 | 14,906 | 5,1817 |
| Events, n (%) | 523 | 1495 | 239 | 568 |
| Person-years | 86,714.4 | 274,668.3 | 91,325.9 | 289,203.5 |
| Crude incidence rate/1000 person years | 6.0 | 5.4 | 2.6 | 2.0 |
| Crude HR (95% CI) | 1.09 (0.99- 1.21) | | 1.31 (1.12-1.52) | |
| P-value | 0.078 | | 0.001 | |
| Adjusted HR (95% CI) | 1.11 (1.00-1.23) | | 1.26 (1.08-1.47) | |
| P-value | 0.046 | | 0.003 | |

CVD = composite CVD, HR= hazard ratio.

Model adjusted for age, Townsend deprivation quintiles, BMI, smoking status, lipid-lowering medication premature delivery, miscarriage, stillbirths, gestational diabetes mellitus, polycystic ovary syndrome, pre-eclampsia, endometriosis.

* = Model adjusted for diabetes mellitus, # = Model adjusted for hypertension

| Source population: women  (N=8,600,835)  Eligible population: 16-50 years, 1995-2018  (N=5,654,155)  ‘Definite’, ‘probable’, & ‘possible’ PID (N=111,790)  Exclude ‘possible’ PID  (N=91986)  ‘Definite’ (N=14231) & ‘probable’ (N=5573) PID cohort  (Total N=19804)  Matched controls  (N=73,769) |
| --- |

Figure S1: Study participant flow chart.

Classifications ‘Definite’, ‘probable’ and ‘possible’ based on French et al.

French CE, Hughes G, Nicholson A, Yung M, Ross JD, Williams T, et al. Estimation of the Rate of Pelvic Inflammatory Disease Diagnoses: Trends in England, 2000–2008. Sex Transm Dis. 2011 Mar;38(3):158–62.
